# The Impact of Bone on the Biology of Aging

**DOI:** 10.1093/geroni/igaa057.2643

**Published:** 2020-12-16

**Authors:** Gerard Karsenty

**Affiliations:** Columbia University, New York, New York, United States

## Abstract

We hypothesized that bone may secrete hormones that regulate energy metabolism and reproduction. Testing this hypothesis revealed that the osteoblast-specific secreted protein osteocalcin is a hormone regulating glucose homeostasis and male fertility by signaling through a GPCR, Gprc6a, expressed in pancreatic β bells and Leydig cells of the testes. The systematic exploration of osteocalcin biology, revealed that it regulates an unexpectedly large spectrum of physiological functions in the brain and peripheral organs and that it has most features of an antigeromic molecule. As will be presented at the meeting, this body of work suggests that harnessing osteocalcin for therapeutic purposes may be beneficial in the treatment of age-related diseases such as depression, age-related memory loss and the decline in muscle function seen in sarcopenia.

